# Cocaine-Related Aortic Dissection: what do we know?

**DOI:** 10.21470/1678-9741-2020-0333

**Published:** 2020

**Authors:** Dustin Greve, Joana Funke, Tiam Khairi, Matteo Montagner, Christoph Starck, Volkmar Falk, Michel Pompeu B. O. Sá, Stephan D. Kurz

**Affiliations:** 1 Department of Cardiovascular Surgery, Charité Universitätsmedizin Berlin, Berlin, Germany.; 2 Department of Cardiothoracic and Vascular Surgery, German Heart Center Berlin, Berlin, Germany.; 3 DZHK (German Centre for Cardiovascular Research), Partner Site, Berlin, Germany.; 4 Department of Health Science and Technology, Swiss Federal Institute of Technology, Zurich, Switzerland.; 5 Division of Cardiovascular Surgery, Pronto-Socorro Cardiológico de Pernambuco (PROCAPE), Universidade de Pernambuco (UPE), Recife, Brazil.

**Keywords:** Aneurysm, Dissection, Cocaine-Related Disorders, Aortic Aneurysm, Thoracic, Vascular Surgical Procedures, Treatment Outcome

## Abstract

**Introduction:**

Cocaine use is known to be associated with an increased risk for vascular diseases. It is likely to trigger or increase the risk for an aortic dissection. We conducted an analysis of 45 cases of cocaine-related aortic dissection to further characterize the clinical features and outcomes of this patient cohort.

**Methods:**

Our study cohort of 45 patients consisted of 11 cases from our institutional database and 34 published case reports.

**Results:**

The observed cases of acute aortic dissection related to cocaine use showed a high proportion of young (41.3±8.67 years) and male (88.9%) patients. Most of the cases (75%) were classified as Stanford type A. Also, in 75% of the cases, cocaine use was prevalent for more than one year. Median time from last cocaine use to onset of symptoms was one hour. In-hospital mortality was 21.4%, while additional 11.9% of the cases died before arriving at the hospital.

**Conclusion:**

Acute aortic dissection related to cocaine use occurs in predominantly young male patients and has a dismal outcome when compared to all comer series.

**Table:** 

Abbreviations, acronyms & symbols	
**IRAD**	**= International Registry of Acute Aortic Dissection**
**NR**	**= Not reported**

## INTRODUCTION

In 2017, the annual prevalence of cocaine use among adults was 2.1% in North America and 1.3% in Europe with increasing numbers over the past few years^[1]^. While the most widespread use of cocaine is through snorting its powder form, the drug is also available as crack cocaine for smoking and intravenous injection. Mortality is assumed to be four to eight times higher for cocaine users as compared to the general population^[2]^. Regular cocaine use is known to be associated with an increased risk of vascular diseases including acute coronary syndrome, myocardial infarction, arrhythmias, and heart failure, as well as cerebrovascular and renovascular disorders^[3-5]^. It has been hypothesized that cocaine use can also increase the risk for acute aortic dissection. Numerous case reports of acute aortic dissection in cocaine users have been reported.

In the International Registry of Acute Aortic Dissection (IRAD), cocaine use was reported in 1.8% of all cases^[6]^. Acute type A aortic dissection occurs at a population-based incidence ranging from 2.1 to 16.3 per 100.000 persons^[7,8]^. The European Union had about 513.5 million inhabitants in 2019. Thus, the number of acute aortic dissections is between 10,784 and 83,701 cases per year. Based on these figures, it could be estimated that aortic dissection occurs in 194-1,507 cocaine users per year. The overall mortality for acute aortic dissection is described by 27.4%^[9]^.

There are various known pathophysiological consequences of cocaine consumption that could increase the risk of aortic dissection. Cocaine stimulates the autonomic nervous system by restraining catecholamine reuptake and expanding affectability of adrenergic nerve endings to norepinephrine^[10,11]^. The autonomic nervous system reacts to cocaine in a similar way as to stress, with an increased heart rate and increased blood pressure^[12,13]^. Cocaine and the effects from alpha agonists show a synergy in terms of vasoconstriction^[14]^. Cocaine is also suspected to cause endothelial dysfunction and have a prothrombotic effect^[15,16]^. The elastic properties of the aorta have been demonstrated to alter in cases of chronic cocaine abuse, showing a decrease in aortic strain and distensibility, as well as a higher aortic stiffness index^[17]^. In addition, the relationship between acute aortic dissection and cocaine use may be modified by other factors such as demographics, lifestyle, or co-use of other drugs. Male gender, smoking, and use of alcohol are the most frequent accompanying risk factors^[18]^. To what extent the risk of acute aortic dissection increases for cocaine users is currently unknown.

We conducted an analysis of 45 cases of cocaine-related aortic dissection to further characterize the clinical features and outcomes of this special patient group.

## METHODS

We gathered data from a sample of patients who suffered an event of acute aortic dissection and reported cocaine use. Aortic dissections of both Stanford types were included. Our study population is comprised of cases from our institutional database and case reports published by March 2020. At the authors’ institution, 11 patients suffered acute aortic dissection related to cocaine use and were merged with the case reports. Hence, 45 patients were eligible for the study. PubMed database was searched for articles with the Medical Subject Headings, or MeSH, terms “Cocaine AND Aortic Dissection” and filtered by case reports. At first, the database search yielded 69 records, screened based on title and abstract. As a result, 36 cases were identified, two of which were excluded due to coronary artery dissection and an intramural hematoma. The list of case reports included in this study is presented in the Supplementary Material section. Cases were reviewed for baseline characteristics, clinical presentation, comorbidities, and outcomes. We also conducted a literature search to find comparable published case series on this topic. Studies were identified using the IBM Watson Analytics: Automating Visualization, Descriptive, and Predictive Statistics software, PubMed, and Google Scholar. As a result, we found five studies to which we compared our results^[6,19-22]^.

## RESULTS

Table 1 shows baseline characteristics of the 45 patients who had an aortic dissection related to the use of cocaine. Approximately three quarters of the patients stated that they had been using cocaine for more than a year. The median time between last cocaine use and symptom onset was one hour. The observed patient cohort showed a strikingly high proportion of young men. In addition to cocaine use, nicotine abuse was present in 84% of the cases. Most of the patients presented with chest pain as primary symptomatology. Although only 57.6% of patients were hypertensive at admission, about three quarters of them had a known history of hypertension. Most of the aortic dissections (75%) were classified as according to Stanford type A. The distribution of surgical and medical therapy regimes is fairly following distribution of Stanford types. The in-hospital mortality was 21.4%. Additional five patients (11.9%) died before arriving at the hospital.

**Table 1:** Characteristics of the study population. Shares are given as "N (%)", measurements are given as "mean (+/- standard deviation)".

N	45
Males	40 (88.9)
Age, years	41.3 (8.67)
**Symptoms on admission**	
Chest pain	23/37 (62.2)
Pain radiating to back	6/23 (26)
Back pain	6/37 (16.2)
Abdominal pain	9/37 (24.3)
Headache	1/37 (2.7)
Dyspnea	4/37 (10.8)
Nausea	6/37 (16.2)
Paresthesia of a limb	4/37 (10.8)
Syncope	2/37 (5.4)
Found dead	5/44 (11.1)
**Blood pressure**	
Known history of hypertension	29/39 (74.4)
Hypertension on admission	19/33 (57.6)
Systolic blood pressure on admission, mmHg	154.13 (45.82)
Diastolic blood pressure on admission, mmHg	91.97 (35.86)
**Substance abuse**	
History of nicotine use	21/25 (84)
Cocaine use for over 1 year	18/25 (72)
Hours from cocaine use to symptom	11 (22.13)
	***(median: 1)***
**Stanford classification**	
Type A	33/44 (75)
Type B	11/44 (25)
**Treatment**	
Surgical	31/39 (79.5)
Medical	8/39 (20.5)
**Outcome**	
Days until discharge	13 (10.67)
	***(median: 9)***
In-hospital mortality	9/42 (21.4)
Pre-hospital mortality	5/42 (11.9)
Days until death	9.25 (13.59)
	***(median: 4)***

A comparison between our study population and five similar published case series of cocaine-related aortic dissection is shown in Table 2. The identified case series range from 13 to 63 patients. Most of the observations from this study group also apply to the previously published case series: young patient age, as well as a high proportion of male patients, and positive smoking and hypertension history. The mortality rate in our study was noticeably higher (21.4% *vs.* 8%) than in the cases from the IRAD published by Dean et al.^[6]^. However, there was a higher proportion of patients with type A aortic dissection in our study population compared to all other studies.

**Table 2:** Comparison of our study cohort with other published case series of aortic dissections and cocaine use.

Characteristics	Daniel et al.^[19]^ (2007)	Dean et al.^[6]^ (2014)	Hsue et al.^[20]^ (2002)	Singh et al.^[21]^ (2007)	Yammine et al.^[22]^ (2019)	Our study
N	16	63	14	13	14	45
Age (years)	47±6.8	47±11	41±8,8	38±9	52.6±7.4	41±8,6
Gender (male)	12 (75%)	55 (87%)	8 (57%)	9 (69%)	10 (71.4%)	40 (88.9%)
History of nicotine abuse	16 (100%)	39 (61%)	14 (100%)	13 (100%)	11 (78.6%)	21/25 (84%)
History of hypertension	11 (69%)	52 (82%)	11 (79%)	9 (69%)	14 (100%)	29/39 (74.4%)
Time from cocaine use to symptoms (mean)	12,8 h (4-24 h)	NR	12 h (0-24 h)	0-48 h	NR	11 h (0-72 h)
Chest pain	NR	51 (81%)	NR	NR	NR	23/37 (62.2%)
Stanford type A	44%	33 (52.4%)	6 (43%)	4 (31%)	0 (0%)	33/44 (75%)
Stanford type B	57%	30 (47.6%)	8 (57%)	9 (69%)	14 (100%)	11/44 (25%)
Surgical treatment	50%	31 (49%)	9 (64%)	-	14 (100%)*	31/39 (79.5%)
Mortality	31% (after 1 year)	8% (in-hospital)	-	69% (after 1 year)	7% (30-day mortality)	9/42 (21.4%) (in-hospital)

NR=not reported

* Endovascular treatment.

## DISCUSSION

Our population of 45 patients with cocaine-related aortic dissection shows great differences compared to ordinary cases of aortic dissection. Compared to the published data from IRAD, this study population was younger (42 *vs.* 61.5 years for type A) and had a higher proportion of men (88.9% *vs.* 66.9%) in comparison to baseline characteristics of all aortic dissection cases^[9]^. The median time from last cocaine use to onset of symptoms in our population was one hour. Cocaine use could therefore trigger the acute event of aortic dissection, possibly based on a previously altered aorta. Bigi et al.^[17]^ demonstrated effects of chronic cocaine use on the elasticity of the aorta which could explain the predisposition to acute aortic dissection. However, in some of the cases in our study, the last cocaine use had no direct temporal connection to the event.

In addition to cocaine use itself, factors such as lifestyle, health behavior, and smoking may play a significant role in the causal relationship to aortic dissections. In all published case series, the proportion of smokers was between 61% and 100%. In our study, history of nicotine abuse was prevalent in 84% of cases. Previous studies also found an increased prevalence of cocaine-related aortic dissection among young, hypertensive, male patients who smoke^[23]^. The prevalence of cocaine use among cases of aortic dissection in the IRAD was 1.8%. The real number may be even higher due to patients not providing truthful information about their drug consumption. A self-reporting method may have introduced a significant bias. The use of urine toxicology screening might help identifying patients who really used cocaine within the past two to four days before the onset of symptoms and those with detectable cocaine metabolites. But nevertheless, drug screening comes with an ethical issue: the willingness of the patients to go through such work-ups that might produce undesired proofs against themselves (*e.g.*, in criminal cases). According to current state of knowledge, it remains unknown how much the risk for an aortic dissection actually increases by cocaine use. We also do not know whether there is a possible relationship between dose and risk.

Because concurrent cocaine use is a relatively rare feature among all aortic dissection cases, patients could be misdiagnosed due to cardiovascular genesis being more likely associated to cocaine use^[4]^. In the history-taking setting of a patient with acute chest pain in the emergency room, the use of drugs should be included in the differential diagnostic considerations and the diagnosis of acute aortic dissection should be considered^[8,24,25]^.

The immediate transport to a cardiac surgery facility is crucial for the further clinical course. There should be no delays here^[26]^.

### Limitations

As our study population only includes 45 cases, the sample may not be representative of all cases of cocaine-related aortic dissection. The cases from our institutional database show an accumulation of white people living in urban regions.

## CONCLUSION

In rare cases, aortic dissections are predisposed or triggered by cocaine use. This special patient group stands out due to a particularly young age and common history of smoking and hypertension. Since the clinical presentation is reminiscent of an acute coronary syndrome, cocaine-related aortic dissections remain a diagnostic challenge.

**Table:** 

Authors' roles & responsibilities	
DG	Substantial contributions to the conception or design of the work; analysis and interpretation of data for the work; agreement to be accountable for all aspects of the work in ensuring that questions related to the accuracy or integrity of any part of the work are appropriately investigated and resolved; final approval of the version to be published
JF	Analysis and interpretation of data for the work; final approval of the version to be published
TK	Analysis and interpretation of data for the work; final approval of the version to be published
MM	Analysis and interpretation of data for the work; final approval of the version to be published
CS	Analysis and interpretation of data for the work; final approval of the version to be published
VF	Analysis and interpretation of data for the work; final approval of the version to be published
MPBOS	Analysis and interpretation of data for the work; final approval of the version to be published
SDK	Substantial contributions to the conception or design of the work; analysis and interpretation of data for the work; agreement to be accountable for all aspects of the work in ensuring that questions related to the accuracy or integrity of any part of the work are appropriately investigated and resolved; final approval of the version to be published

## SUPPLEMENTARY MATERIAL List of included case reports:

1. Adkins MS, Gaines WE, Anderson WA, Laub GW, Fernandez J, McGrath LB. Chronic type A aortic dissection: an unusual complication of cocaine inhalation. Ann Thorac Surg. 1993;56(4):977-9. doi:10.1016/0003-4975(93)90372-o.

2. Barth CW 3rd, Bray M, Roberts WC. Rupture of the ascending aorta during cocaine intoxication. Am J Cardiol. 1986;57(6):496. doi:10.1016/0002-9149(86)90787-3.

3. Baumgartner FJ, Omari BO. Method of repair of cocaine-induced chronic type A aortic dissection. Ann Thorac Surg. 1997;64(5):1518-9.

4. Chang RA, Rossi NF. Intermittent cocaine use associated with recurrent dissection of the thoracic and abdominal aorta. Chest. 1995;108(6):1758-62. doi:10.1378/chest.108.6.1758.

5. Cohle SD, Lie JT. Dissection of the aorta and coronary arteries associated with acute cocaine intoxication. Arch Pathol Lab Med. 1992;116(11):1239-41.

6. Correale M, Di Martino L, Ieva R, Di Biase M, Brunetti ND. Aortic dissection after cocaine abuse. Clin Res Cardiol. 2011;100(12):1129-30. doi:10.1007/s00392-011-0363-7.

7. Croft AP, Nader K, Arulanantham N. A very unusual headache. Clin Med (Lond). 2014;14(1):58-60. doi:10.7861/clinmedicine.14-1-58.

8. Dabbagh A, Arabnia MK, Foroughi M, Shahzamani M, Rahmian H. The use of intraoperative transesophageal echocardiography in thoracic aortic dissection due to chronic cocaine abuse. Anesth Pain Med. 2016;7(1):e35254. doi:10.5812/aapm.35254.

9. Davis CB, Kendall JL. Emergency bedside ultrasound diagnosis of superior mesenteric artery dissection complicating acute aortic dissection. J Emerg Med. 2013;45(6):894-6. doi:10.1016/j.jemermed.2013.04.025.

10. D'Errico S, Niballi S, Bonuccelli D. Aortic dissection in cocaine abuse: a fatal case. J Forensic Leg Med. 2018;58:179-82. doi:10.1016/j.jflm.2018.07.001.

11. Dewar K, Nolan S. Chronic hypertension, recreational cocaine use and a subsequent acute aortic dissection in a young adult. BMJ Case Rep. 2017;2017:bcr2016218235. doi:10.1136/bcr-2016-218235.

12. Dobrilovic N, Stockwell PH, Singh AK. Valve-sparing aortic root replacement after cocaine-induced acute ascending aortic dissection. J Card Surg. 2011;26(2):207-10. doi:10.1111/j.1540-8191.2010.01191.x.

13. Edwards J, Rubin RN. Aortic dissection and cocaine abuse. Ann Intern Med. 1987;107(5):779-80. doi:10.7326/0003-4819-107-5-779_2.

14. Famularo G, Polchi S, Di Bona G, Manzara C. Acute aortic dissection after cocaine and sildenafil abuse. J Emerg Med. 2001;21(1):78-9. doi:10.1016/s0736-4679(01)00345-6.

15. Fisher A, Holroyd BR. Cocaine-associated dissection of the thoracic aorta. J Emerg Med. 1992;10(6):723-7. doi:10.1016/0736-4679(92)90532-x.

16. Gadaleta D, Hall MH, Nelson RL. Cocaine-induced acute aortic dissection. Chest. 1989;96(5):1203-5. doi:10.1378/chest.96.5.1203.

17. Grannis FW Jr, Bryant C, Caffaratti JD, Turner AF. Acute aortic dissection associated with cocaine abuse. Clin Cardiol. 1988;11(8):572-4. doi:10.1002/clc.4960110811.

18. Hohm SP. A 28-year-old man with an aortic dissection and history of cocaine abuse. J Emerg Nurs. 1995;21(3):199-201. doi:10.1016/s0099-1767(05)80151-5.

19. Madu EC, Shala B, Baugh D. Crack-cocaine-associated aortic dissection in early pregnancy--a case report. Angiology. 1999;50(2):163-8. doi:10.1177/000331979905000212.

20. Marella GL, Furnari C, Perfetti E, Arcudi G. Aortic dissection and cocaine use. J Forensic Leg Med. 2011;18(7):329-31. doi:10.1016/j.jflm.2011.06.008.

21. McDermott JC, Schuster MR, Crummy AB, Acher CW. Crack and aortic dissection. Wis Med J. 1993;92(8):453-5.

22. Mohamed MA, Abraham R, Maraqa TI, Elian S. Cocaine-induced type-A aortic dissection extending to the common iliac arteries. Cureus. 2018;10(1):e2059. doi:10.7759/cureus.2059.

23. Nallamothu BK, Saint S, Kolias TJ, Eagle KA. Clinical problem-solving. Of nicks and time. N Engl J Med. 2001;345(5):359-63. doi:10.1056/NEJM200108023450508.

24. Nusair M, Abuzetun JY, Khaja A, Dohrmann M. A case of aortic dissection in a cocaine abuser: a case report and review of literature. Cases J. 2008;1(1):369. doi:10.1186/1757-1626-1-369.

25. Om A, Porter T, Mohanty PK. Transesophageal echocardiographic diagnosis of acute aortic dissection complicating cocaine abuse. Am Heart J. 1992;123(2):532-4.

26. Palmiere C, Burkhardt S, Staub C, Hallenbarter M, Paolo Pizzolato G, Dettmeyer R, et al. Thoracic aortic dissection associated with cocaine abuse. Forensic Sci Int. 2004;141(2-3):137-42. doi:10.1016/j.forsciint.2003.12.018.

27. Perron AD, Gibbs M. Thoracic aortic dissection secondary to crack cocaine ingestion. Am J Emerg Med. 1997;15(5):507-9. doi:10.1016/s0735-6757(97)90196-0.

28. Rashid J, Eisenberg MJ, Topol EJ. Cocaine-induced aortic dissection. Am Heart J. 1996;132(6):1301-4. doi:10.1016/s0002-8703(96)90486-x.

29. Riaz K, Forker AD, Garg M, McCullough PA. Atypical presentation of cocaine-induced type A aortic dissection: a diagnosis made by transesophageal echocardiography. J Investig Med. 2002;50(2):140-2. doi:10.2310/6650.2002.31309.

30. Shah R, Berzingi C, Fan TH, Askari R, Khan MR. Cocaine-induced acute aortic dissection. J Emerg Med. 2015;49(3):e87-9. doi:10.1016/j.jemermed.2015.02.006.

31. Sherzoy A, Sadler D, Brown J. Cocaine-related acute aortic dissection diagnosed by transesophageal echocardiography. Am Heart J. 1994;128(4):841-3. doi:10.1016/0002-8703(94)90289-5.

32. Simons AJ, Arazoza E, Hare CL, Smulyan H, Lighty GW Jr, Parker FB Jr. Circumferential aortic dissection in a young woman. Am Heart J. 1992;123(4 Pt 1):1077-9. doi:10.1016/0002-8703(92)90727-d.

33. Volman MN, Schoch AG, van Leeuwen RP, Tukkie R. Haematemesis, abdominal pain and a diastolic murmur in a cocaine user. Stanford type-A aortic dissection. Neth J Med. 2010;68(12):422, 426-7.
